# Enzymatic indicators reveal drought sensitivity of the deadwood–soil system in temperate forests

**DOI:** 10.1038/s41598-026-54208-6

**Published:** 2026-05-23

**Authors:** Adam Górski, Jarosław Lasota, Ewa Błońska

**Affiliations:** Department of Ecology and Silviculture, Faculty of Forestry, University of Agriculture in Krakow, 29 Listopada 46 Str., 31-425 Kraków, Poland

**Keywords:** Decomposition process, Deadwood, Enzymes, Moisture, Soil, Drought, Forest ecosystem, Ecology, Ecology, Environmental sciences, Plant sciences

## Abstract

Drought can disrupt biogeochemical functioning in forest ecosystems by limiting microbial activity and extracellular enzyme production. We investigated the effects of simulated moisture deficit on the activity of five enzymes involved in C, N and P acquisition in a deadwood–soil system: β-glucosidase (BG), β-D-cellobiosidase (CB), β-xylosidase (XYL), N-acetyl-β-D-glucosaminidase (NAG) and phosphatase (PH). Deadwood of six temperate tree species (broadleaf and conifer) was exposed to drought and control conditions over two years (2023–2024), and enzyme activities were measured seasonally in both deadwood and the underlying soil. Drought conditions led to a pronounced reduction in the activity of all analyzed enzymes in deadwood, frequently exceeding 50% compared to control treatments, which indicates strong moisture limitation of microbial processes during wood decomposition. Enzymatic activity in soil beneath deadwood also decreased, although the smaller absolute changes observed in soil are likely related to lower initial activity levels rather than increased resistance to drought. The extent of decline varied among enzymes and wood species, with β-glucosidase and β-xylosidase showing differences of approximately 30–40% between treatments in soil, and with some species (e.g. beech and spruce) exhibiting weaker responses to drought. Enzyme activity in both substrates followed a consistent seasonal pattern, with maxima in summer and minima in winter. The increasing divergence between control and drought treatments in the second-year highlights cumulative effects of prolonged water limitation. The results demonstrate that extracellular enzyme activity is highly sensitive to moisture availability and that deadwood and underlying soil respond differently to drought. This provides mechanistic insight into how drought may alter microbial functioning and decomposition dynamics at the deadwood–soil interface in forest ecosystems. General Linear Models, correlation analysis and PCA consistently indicated moisture as a major factor associated with enzymatic variation, whereas temperature showed no significant relationships with enzyme activities. Reduced enzyme activity under drought suggests a limitation of microbial decomposition processes and nutrient acquisition, particularly for carbon-, nitrogen- and phosphorus-related pathways, potentially constraining microbial metabolism and slowing organic matter turnover at the deadwood–soil interface.

**Supplementary Information:**

The online version contains supplementary material available at 10.1038/s41598-026-54208-6.

## Introduction

Wood decomposition is a fundamental process driving the cycling of matter and energy in terrestrial ecosystems^1,2^. Deadwood plays a key role in forest ecosystems by providing habitat for diverse organisms and contributing to soil organic matter formation and long-term soil stability. As decomposition progresses, it becomes a hotspot of biological activity, where fungi and bacteria break down structural components and drive the cycling of essential elements such as carbon, nitrogen, and phosphorus within the soil–wood system^3^. This process is mediated by microbial enzymes that degrade cellulose, hemicellulose, and lignin into simpler compounds, which can be transferred to the soil, enhancing nutrient availability and overall ecosystem functioning^4^. Enzymatic activity, which governs the efficiency of decomposition, is strongly shaped by both biotic and abiotic factors, particularly environmental conditions^5,6^. Soil microorganisms are the main producers of enzymes responsible for decomposition^7^. Species diversity, abundance and activity of microorganisms determine the intensity of enzymatic processes^8^. Substrate quality and chemical composition, in both soil and wood, also play a crucial role in regulating enzyme activity^9^. Among abiotic drivers, water availability is particularly important, as it regulates enzyme diffusion, substrate accessibility, and microbial metabolism^10^. Drought strongly suppresses enzymatic activity^11^. Other environmental constraints, including pH, nutrient availability and temperature-related stress, also influence microbial enzyme production^11,12^. Drought leads to a decrease in the metabolic activity of microorganisms and a reduction in their ability to produce enzymes^13^. Lack of water limits the diffusion of enzymes and substrates, a phenomenon that is particularly relevant in forest soils and decomposing wood. Under drought conditions, enzymatic activity and microbial metabolism significantly decrease, affecting processes such as organic matter mineralization and nitrogen transformation^14^. Drought inhibits the mobility of many nutrients, such as nitrogen, phosphorus and potassium^15^. Water deficiency further restricts nutrient transport in the soil, limiting their availability to organisms. Drought can also cause stress in microorganisms, which leads to a reduction in their ability to synthesize enzymes^16^. Long-term drought leads to a decrease in the diversity of microorganisms and soil fauna^17^. Although the strong influence of moisture on enzymatic activity is well established, no studies have examined enzymatic activity simultaneously in the soil–deadwood system under conditions of experimentally simulated drought. Therefore, investigating enzyme responses in this coupled system provides new insights into decomposition processes and nutrient cycling under water-limited conditions, particularly in forest ecosystems where soil and deadwood interactions play a key role.

The aim of this study was to determine the impact of simulated drought on the activity of enzymes involved in deadwood decomposition and to compare these responses with enzymatic activity in soils directly influenced by decaying wood. By simultaneously examining enzymatic responses in both deadwood and the associated soil, this study will contribute to improving our understanding of drought effects on the coupled wood–soil system, which plays an important role in forest biogeochemical cycling and soil functional stability. Despite increasing interest in deadwood decomposition, most studies have focused either on deadwood or soil separately. The coupled deadwood–soil system remains poorly understood, particularly in terms of how biogeochemical processes operate across this interface. This is a critical gap, as decomposing wood and underlying soil form a functional continuum that regulates nutrient transfer and microbial activity. Unlike previous studies focusing solely on soil or individual wood species, our experiment integrates six contrasting tree species, enabling a direct comparison of drought sensitivity between hardwood and softwood species across multiple enzyme pathways. The selected species represent key functional groups of temperate forests, spanning a broad gradient of wood density, lignin content, and anatomical structure known to influence decomposition processes. This experimental design allowed us to compare drought sensitivity among contrasting wood substrates and functional groups rather than focusing on a single species or substrate type. The two-year monitoring under controlled drought conditions provides a rare dataset capturing cumulative, long-term biochemical responses of the wood–soil system to moisture deficit. We hypothesized that drought would reduce the activity of key enzymes involved in the carbon (C), nitrogen (N), and phosphorus (P) cycles, with stronger and progressively increasing effects in deadwood compared to soil. Understanding how drought affects biochemical processes in the wood–soil system is important for improving predictions of decomposition dynamics and nutrient cycling under changing moisture conditions.

## Materials and methods

### Study area and experiment design

In January 2023, a simulated drought experiment was initiated. The study was conducted near Miechów, southern Poland (50.3740° N, 19.9807° E). The soil at the study site was classified as Typic Hapludalfs (Alfisols), characterized by a well-developed Bt illuvial horizon, moderate base saturation, and a slightly acidic reaction (pH 6.0–6.5). The soil exhibited a relatively high clay content, with notable concentrations of iron and aluminum oxides and trace amounts of calcium carbonate, consistent with Alfisols typical of humid temperate regions. The experimental plot was carefully selected to ensure topographic homogeneity, minimizing the influence of microrelief on soil moisture redistribution. The experiment was conducted in the forest nursery of the Miechów Forest District, within a section of the nursery located beneath the canopy of an existing forest stand. The study design assumed comparable background environmental conditions, particularly with respect to soil properties, organic matter inputs, and light exposure. A flat, vegetation-free surface was deliberately chosen to avoid confounding effects of surface runoff, lateral water movement, and plant water uptake, which could otherwise compromise the comparability between the control and drought treatments.

**Fig. 1:**
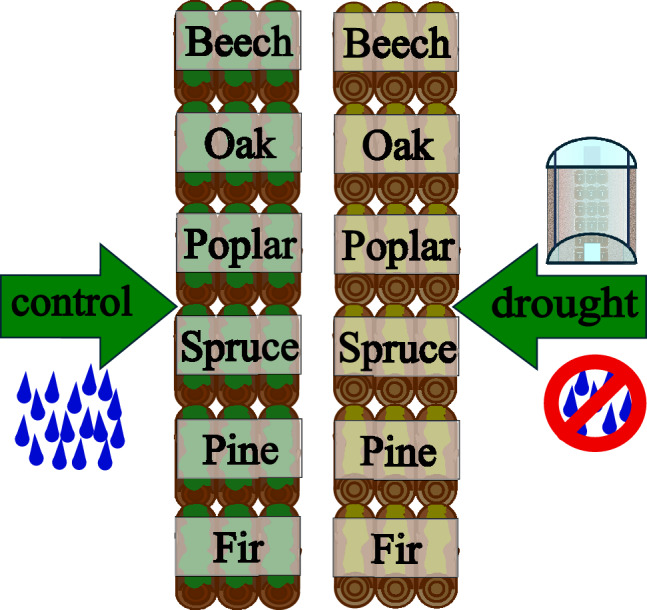
Schematic representation of the experimental design showing the distribution of deadwood logs across control and drought treatments. Six tree species were included in each treatment, with three replicate logs per species. The control treatment was exposed to natural precipitation, whereas the drought treatment was placed under a rain-exclusion structure. Figure created by the authors.

To simulate drought conditions, a rain-exclusion system constructed from transparent polycarbonate panels was installed above the experimental area. The structure had fully open lateral sides, ensuring natural air circulation and preventing artificial temperature accumulation beneath the cover. The system was designed solely to exclude direct atmospheric precipitation, without modifying ambient temperature or radiation regimes. Logs assigned to the drought treatment (DD) were placed beneath this panel-covered structure, whereas control logs (DC) were placed in an adjacent open area without any frame or cover, thus remaining fully exposed to natural precipitation (Fig. 1). Deadwood from six tree species was included in the experiment: pedunculate oak (*Quercus robur*), aspen (*Populus tremula*), common beech (*Fagus sylvatica*), silver fir (*Abies alba*), Norway spruce (*Picea abies*), and Scots pine (*Pinus sylvestris*). For the entire experiment, 36 logs were used in total (2 treatment variants × 6 species × 3 replicates), with three logs per species assigned to each treatment. In the drought treatment, all 18 logs (6 species × 3 replicates) were placed beneath a single rain-exclusion structure, while in the control treatment, the corresponding 18 logs (6 species × 3 replicates) were placed together in an adjacent open area exposed to natural precipitation. These species were selected to represent dominant broadleaf and conifer functional groups of temperate forests, encompassing a broad gradient of wood density, chemical composition, and anatomical structure known to influence decomposition processes. The wood material was collected in situ from forest stands and transported to the experimental site. All logs were classified as decay class IV according to the classification of Maser et al.^18^, indicating advanced decomposition. This decay stage was intentionally selected because it represents a phase in which microbial activity is high and wood structure is substantially altered, making it particularly sensitive to changes in moisture availability. As a result, logs at this stage are well suited for assessing drought effects on enzymatic processes. Each species was represented by three logs per treatment, each approximately 50 cm in length and exceeding 30 cm in diameter at the thinner end, with a taper of less than 1 cm per meter. All remaining remnants of branches were removed to standardize surface conditions, and logs of comparable size and decomposition stage were selected.

Temperature and moisture conditions of the substrate were monitored in both drought and control treatments. Measurements were conducted using 5TM sensors (Decagon Devices, Pullman, WA, USA), which record temperature and volumetric water content directly within the measured material. Sensors were installed in the soil and inserted directly into the deadwood to continuously monitor substrate-specific microclimatic conditions at one-hour intervals throughout the experiment. Samples for laboratory analyses were collected eight times during the study period, specifically in March, June, September, and December of 2023 and 2024. In the analysis, four types of samples were distinguished, including deadwood (D) and soil (S), collected from both control plots (c) and drought-affected plots (d). Based on this classification, four sample categories were defined: Dc, Dd, Sc, and Sd. This approach allows for the simultaneous assessment of the effects of both substrate type and environmental conditions on the analyzed parameters. Wood samples were collected from the wood–soil contact zone, using a 1.8 cm diameter auger, drilling approximately 5 cm from the outer surface toward the interior of each log. The resulting wood chips were then ground following standard laboratory procedures and homogenized prior to enzymatic analysis. Soil samples were collected directly beneath each log using a metal sampler from the 0–10 cm mineral soil layer. All soil samples were taken from a central position beneath each log to ensure consistency and minimize spatial variability associated with edge effects.

### Laboratory analysis

Soil samples were sieved through a 2 mm mesh prior to enzymatic activity analysis to ensure homogenization. The enzyme activities were determined using fluorogenically labeled substrates in a microplate assay, following established fluorometric procedures described by Pritsch et al.^19^, Turner^20^, and Sanaullah et al.^21^. The fluorogenic enzyme substrates based on 4-methylumbelliferone (MUB) were used: MUB-β-D-cellobioside for β-D-cellobiosidase (CB), MUB-β-D-xylopyranoside for β-xylosidase (XYL), MUB-N-acetyl-β-D-glucosaminide for N-acetyl-β-D-glucosaminidase (NAG), MUB-β-D-glucopyranoside for β-glucosidase (BG) and MUB-phosphate for phosphatase (PH). The assay conditions applied in this study, including buffer-based incubation and fluorescence detection of MUB-linked substrates, were consistent with these previously published protocols and with procedures previously used in our laboratory for enzymatic analyses^9,22,23^. To ensure comparability across all samples, the same incubation conditions and substrate addition were applied within each assay. A 2.75 g subsample of soil or wood (deadwood was shredded using a laboratory grinder prior to analysis) was mixed with 92 mL of modified universal buffer (pH = 6.0). The suspensions were pipetted into wells of a 96-well microplate containing the substrate and buffer solution. Fluorescence was determined by first incubating the soil and wood suspensions for 1.5 hours at 35 °C in 96-well microplates (Puregrade, Germany) and then measuring with fluorogenic substrates at an excitation wavelength of 355 nm and an emission wavelength of 460 nm. The measurement was performed using a Synergy HTX BioTek plate reader. The substrate was added in the same amount to each 0.2 mL assay mixture. In parallel with the determination of enzymatic activity, the moisture content of the samples was measured using the oven-dry method. Moisture measurements were carried out concurrently with each sampling event throughout the experiment. Enzymatic activity was expressed in nmol MUB g^−1^ ·h^−1^ and was calculated on a dry weight basis of the analyzed material.

### Statistical analysis

Spearman’s correlation coefficient was employed to evaluate the relationships between enzyme activity and factors such as temperature and moisture. The impact of various factors (wood species, sample type, and moisture condition) on enzyme activity in wood and soil was analyzed using General Linear Models (GLM). In addition to the main effects, the models included selected interaction terms among the analyzed factors. This approach was used to identify general patterns of combined factor effects in the dataset. Because the study was based on repeated seasonal observations within the same experimental system, the GLM results were interpreted as describing general patterns in the dataset rather than a fully hierarchical repeated-measures structure. Prior to analysis, the dataset was screened for obvious data-entry errors and extreme values, and variable distributions were inspected to support the interpretation of the statistical results. Principal Component Analysis (PCA) was performed to explore the relationships among the measured variables and to identify patterns in enzyme activity and environmental conditions across samples. The analysis included enzyme activities (BG, CB, NAG, XYL, and phosphatase), as well as physicochemical parameters such as moisture and temperature. Prior to PCA, all variables were standardized (centred and scaled to unit variance) to account for differences in measurement units and to ensure comparability among variables. PCA was conducted based on the correlation matrix, and the first two principal components were retained for interpretation, as they explained the largest proportion of total variance in the dataset. The ordination biplot was used to visualize both the distribution of samples and the direction and strength of relationships among variables. The orientation and length of vectors in the biplot were used to interpret correlations among variables and their association with specific sample groups. A significance threshold of p < 0.05 was set. All statistical analyses were performed using the R programming language^24^ in the RStudio environment^25^. The readxl^26^, dplyr^27^ and ggplot2^28^ packages were used to prepare graphs with enzymatic activity. The corrplot package^29^ was used to create the correlation plot, whereas the MASS^30^, factoextra^31^ and ggfortify^32^ packages were used for PCA and its visualization.

## Results

**Fig. 2:**
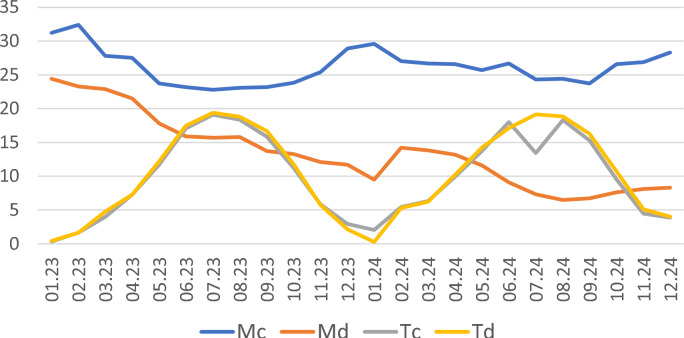
Average monthly soil moisture and soil temperature in the control and drought treatments during the study period (January 2023–December 2024). Mc, soil moisture in the control treatment; Md, soil moisture in the drought treatment; Tc, soil temperature in the control treatment; Td, soil temperature in the drought treatment. The x-axis shows month and year; the y-axis shows soil moisture (%) and soil temperature (°C).

Substrate moisture was significantly lower under drought compared to control conditions, with an average difference of approximately 13% between control and drought, indicating a marked reduction in water availability (Fig. 2). Toward the end of the experiment, this difference increased to nearly 20%, reflecting the cumulative effect of prolonged drought. Substrate temperature (soil and deadwood) did not differ substantially between treatments (Fig. 2), with mean values of 9.8 °C in the control and 10.2 °C in the drought variant, suggesting that the observed enzymatic responses were more closely associated with moisture differences than with temperature differences. Higher values were consistently observed in decomposing wood under the control treatment (DC) compared to the drought treatment (DD) throughout the study period (Fig. 1S).

**Fig. 3:**
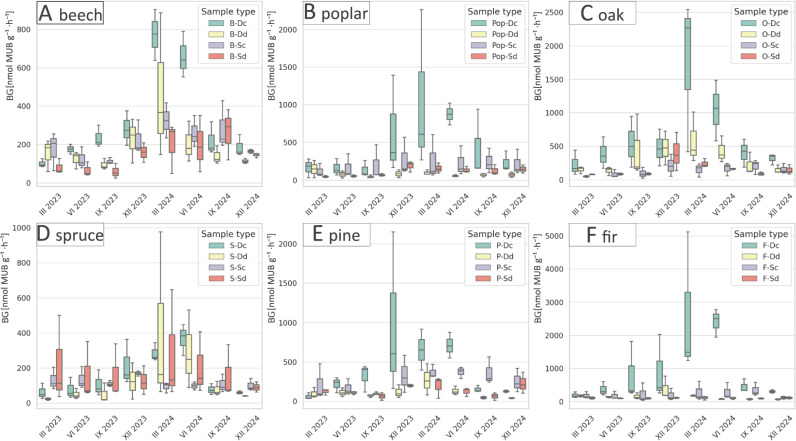
Seasonal variation in β-glucosidase (BG) activity in deadwood and underlying soil under control and drought treatments. Panels correspond to tree species: (**A**) *Fagus sylvatica*, (**B**) *Populus tremula*, (**C)** *Quercus robur*, (**D**) *Picea abies*, (**E**) *Pinus sylvestris*, and (**F**) *Abies alba*. Sample category labels combine species identity with sample type and treatment: D-c, deadwood under control; D-d, deadwood under drought; S-c, soil under control; and S-d, soil under drought. The x-axis shows sampling dates from March 2023 to December 2024, and the y-axis shows enzyme activity (nmol MUB g^−1^ ·h^−1^).

Under control conditions, the highest β-glucosidase (BG) activity in deadwood was observed in beech (B) and oak (O), with pronounced peaks occurring in March and June, particularly in 2024 (Fig. 3). Coniferous species (pine and spruce) consistently exhibited lower BG activity during the same periods. BG activity declined markedly during winter, particularly in December, across all wood species. Beech, spruce and fir showed a winter reduction of 17–24% in December 2024, confirming a strong seasonal constraint on enzymatic activity. BG activity in soil was substantially lower than in deadwood in both treatments. Under drought conditions, BG activity in deadwood was reduced by approximately 50% relative to the control, while soil activity remained consistently lower than wood across all sampling dates (Fig. 3).

**Fig. 4:**
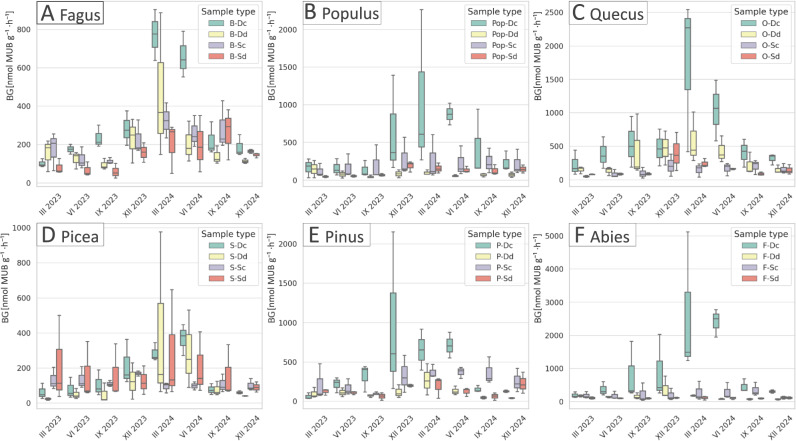
Seasonal variation in β-D-cellobiosidase (CB) activity in deadwood and underlying soil under control and drought treatments. Panels correspond to tree species: (**A**) *Fagus sylvatica*, (**B**) *Populus tremula*, (**C**) *Quercus robur*, (**D**) *Picea abies*, (**E**) *Pinus sylvestris*, and (**F**) *Abies alba*. Sample category labels combine species identity with sample type and treatment: D-c, deadwood under control; D-d, deadwood under drought; S-c, soil under control; and S-d, soil under drought. The x-axis shows sampling dates from March 2023 to December 2024, and the y-axis shows enzyme activity (nmol MUB g^−1^ ·h^−1^).

Under control conditions, β-D-cellobiosidase (CB) activity was generally higher in deadwood than in soil, although the magnitude of this difference varied across sampling periods and between years, with the most pronounced contrasts observed in 2024 (Fig. 4). Drought reduced CB activity in both substrates, with a stronger decline observed in deadwood. Broadleaf species (beech and oak) generally exhibited higher mean CB activity than conifers. Seasonal declines between September and December within each year (2023 and 2024) were evident, with reductions ranging from 4–45% in deadwood and 10–54% in soil, depending on species. The drought effect intensified in the second year (2024), when mean CB activity declined by approximately 40% in deadwood, compared to a relatively small decrease (~3%) in soil (Fig. 4).

**Fig. 5:**
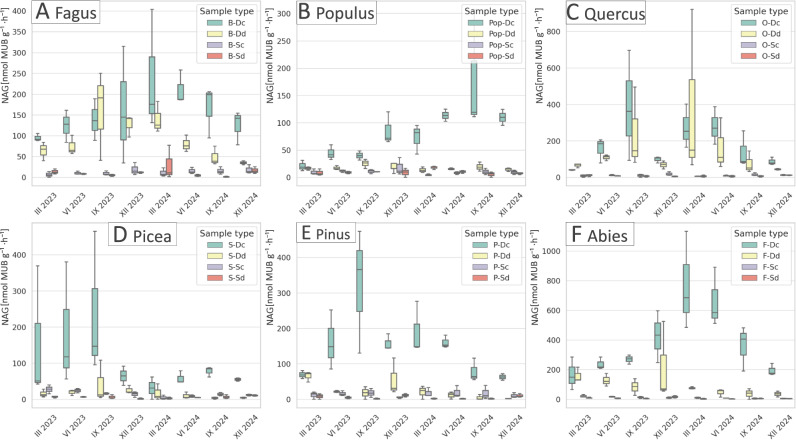
Seasonal variation in N-acetyl-β-D-glucosaminidase (NAG) activity in deadwood and underlying soil under control and drought treatments. Panels correspond to tree species: (**A**) *Fagus sylvatica*, (**B**) *Populus tremula*, (**C**) *Quercus robur*, (**D**) *Picea abies*, (**E**) *Pinus sylvestris*, and (**F**) *Abies alba*. Sample category labels combine species identity with sample type and treatment: D-c, deadwood under control; D-d, deadwood under drought; S-c, soil under control; and S-d, soil under drought. The x-axis shows sampling dates from March 2023 to December 2024, and the y-axis shows enzyme activity (nmol MUB g^−1^ ·h^−1^).

Under control conditions, NAG activity was generally higher in wood than in soil (Fig. 5). Drought reduced enzyme activity in both sample types, although the magnitude of this effect varied among species and was not consistently greater in soil. Beech and oak wood showed high NAG activity, particularly in June and September under control conditions, while fir exhibited the highest activity in deadwood during the second year. As with CB, NAG activity peaked in summer and declined toward winter. Drought caused an additional reduction of approximately 15–20% in deadwood between September and December (Fig. 5).

**Fig. 6:**
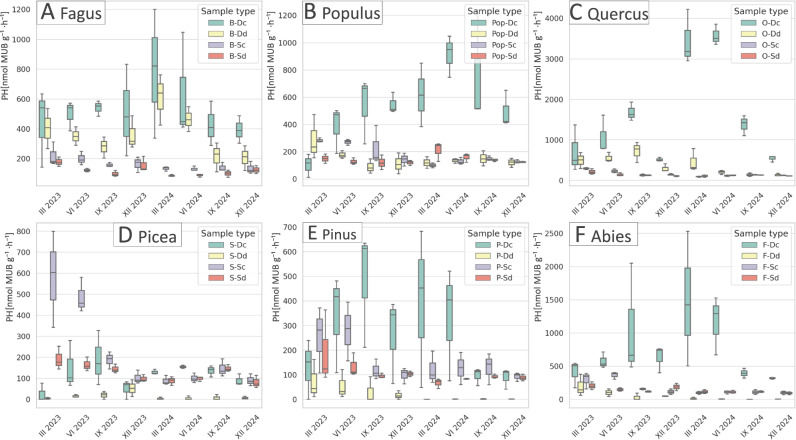
Seasonal variation in phosphatase (PH) activity in deadwood and underlying soil under control and drought treatments. Panels correspond to tree species: (**A**) *Fagus sylvatica*, (**B**) *Populus tremula*, (**C**) *Quercus robur*, (**D**) *Picea abies*, (**E**) *Pinus sylvestris*, and (**F**) *Abies alba*. Sample category labels combine species identity with sample type and treatment: D-c, deadwood under control; D-d, deadwood under drought; S-c, soil under control; and S-d, soil under drought. The x-axis shows sampling dates from March 2023 to December 2024, and the y-axis shows enzyme activity (nmol MUB g^−1^ ·h^−1^).

Under control conditions, phosphatase was more active in wood than in soil (Fig. 6). Drought reduced phosphatase activity by about 50%, with substantial declines observed in both wood and soil. Phosphatase activity showed a clear seasonal pattern, with the highest values generally recorded in summer (June, September), and low activity in winter across all samples, particularly under drought conditions (Fig. 6). Relatively high activity was also observed in March 2024 for many control samples.

**Fig. 7:**
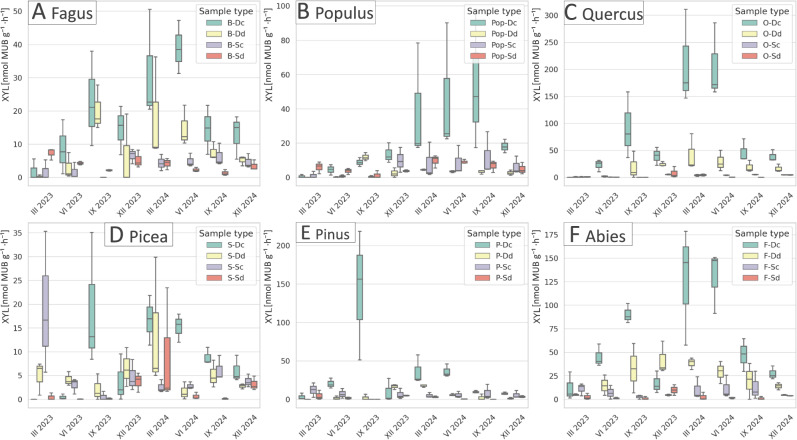
Seasonal variation in β-xylosidase (XYL) activity in deadwood and underlying soil under control and drought treatments. Panels correspond to tree species: (**A**) *Fagus sylvatica*, (**B**) *Populus tremula*, (**C**) *Quercus robur*, (**D**) *Picea abies*, (**E**) *Pinus sylvestris*, and (**F**) *Abies alba*. Sample category labels combine species identity with sample type and treatment: D-c, deadwood under control; D-d, deadwood under drought; S-c, soil under control; and S-d, soil under drought. The x-axis shows sampling dates from March 2023 to December 2024, and the y-axis shows enzyme activity (nmol MUB g^−1^ ·h^−1^).

XYL activity was higher in wood than in soil in both variants. The strongest contrast occurred in beech (B) and oak (O) under control conditions, where soil activity was 79–96% lower than in wood (Fig. 7). Drought caused a clear decrease in activity, especially in soil. The highest XYL activity values were recorded in the summer months of 2023 and 2024, while activity decreased markedly in winter, with drought causing an additional reduction of approximately 20–30% in wood (September → December). (Fig. 7).

Table 1 presents the results of the General Linear Models (GLM) evaluating the effects of wood species (Sp), sample type (ST), moisture content (M), and seasonality (S) on enzyme activity. All studied factors had a significant effect on the enzymatic activities of β-glucosidase (BG), β-D-cellobiosidase (CB), N-acetyl-β-D-glucosaminidase (NAG), phosphatase (PH) and β-xylosidase (XYL) (p < 0.05). For example, moisture content (M) had the highest F values ​​(e.g. 71.01 for phosphatase) indicating a particularly strong effect on enzyme activity. Significant interaction effects were observed between species and sample type, as well as between species and moisture, indicating that enzymatic activity responses depended on both substrate and environmental conditions. In contrast, interactions involving seasonality were generally not significant (p > 0.05), suggesting that higher-order interactions involving seasonality were not strongly expressed in the dataset.

**Table 1:** GLM analysis results for enzyme activity significance effect (p < 0.05) are shown in bold; β-glucosidase (BG), β-D-cellobiosidase (CB), N-acetyl-β-D-glucosaminidase (NAG), phosphatase (PH), β-xylosidase (XYL).

	BG		CB		NAG		PH		XYL	
	F	p	F	p	F	p	F	p	F	p
Species (Sp)	5.26	**<0.01**	6.78	**<0.01**	12.63	**<0.01**	17.92	**<0.01**	10.64	**<0.01**
Sample type (ST)	32.84	**<0.01**	49.96	**<0.01**	185.17	**<0.01**	64.81	**<0.01**	84.54	**<0.01**
Moisture (M)	46.52	**<0.01**	42.15	**<0.01**	51.31	**<0.01**	71.01	**<0.01**	43.01	**<0.01**
Seasonality (S)	11.15	**<0.01**	8.66	**<0.01**	3.24	**<0.01**	3.51	**<0.01**	7.90	**<0.01**
Sp*ST	4.77	**<0.01**	4.77	**<0.01**	9.12	**<0.01**	15.71	**<0.01**	8.98	**<0.01**
Sp* M	4.79	**<0.01**	4.52	**<0.01**	3.65	**<0.01**	6.17	**<0.01**	3.24	**<0.01**
Sp* S	0.91	0.64	1.12	0.29	0.82	0.74	1.05	0.37	1.28	0.13
Sp*ST*M*S	0.84	0.72	1.24	0.15	1.02	0.43	1.32	0.11	1.17	0.22

**Fig. 8:**
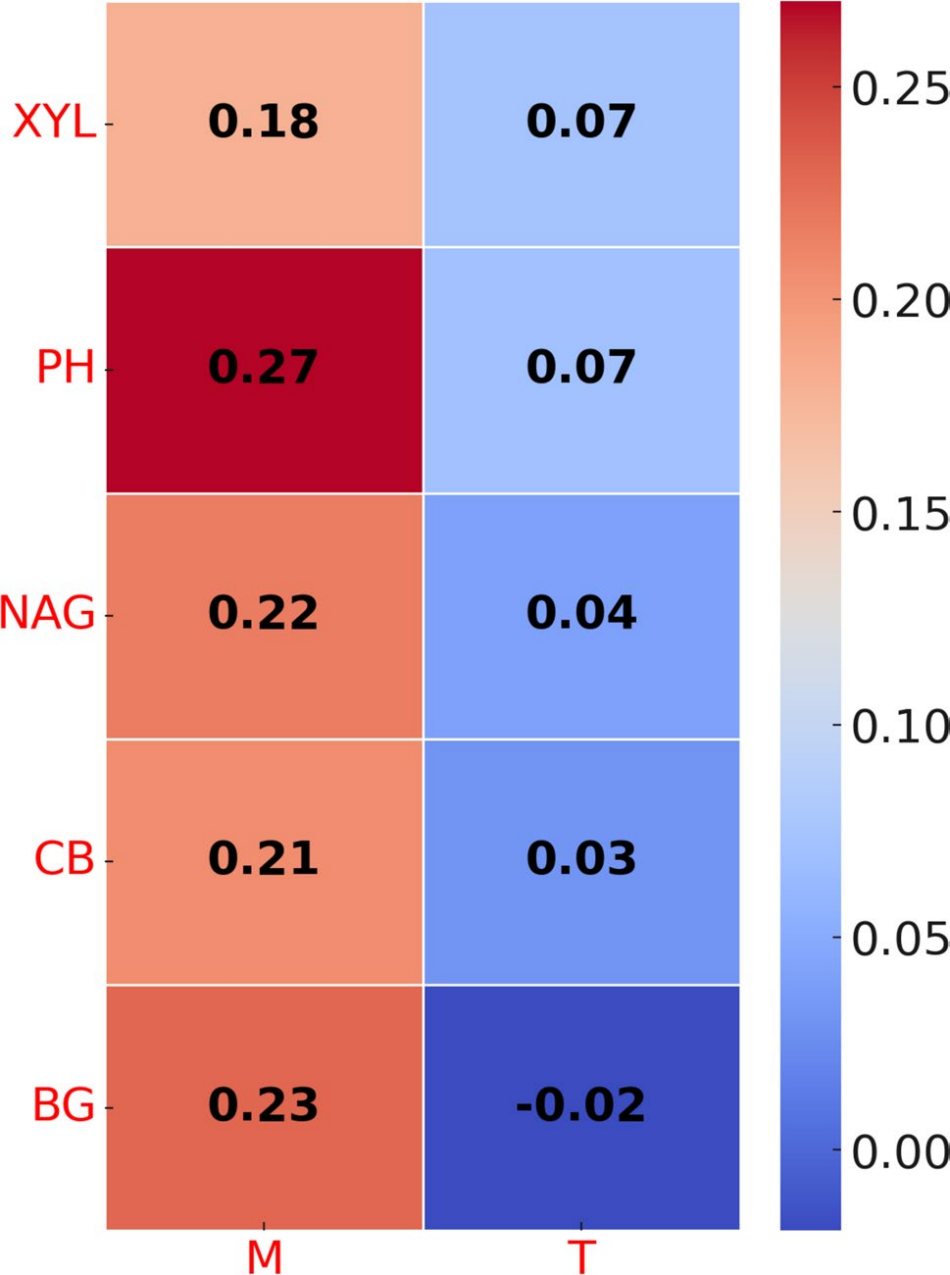
Spearman correlation coefficients between enzyme activity and moisture (M) and temperature (T). The heatmap shows correlation coefficients for β-xylosidase (XYL), phosphatase (PH), N-acetyl-β-D-glucosaminidase (NAG), β-D-cellobiosidase (CB), and β-glucosidase (BG), with warmer colours (shades of red) indicating stronger positive correlations and cooler colours (shades of blue) indicating weaker or negative correlations.

**Fig. 9:**
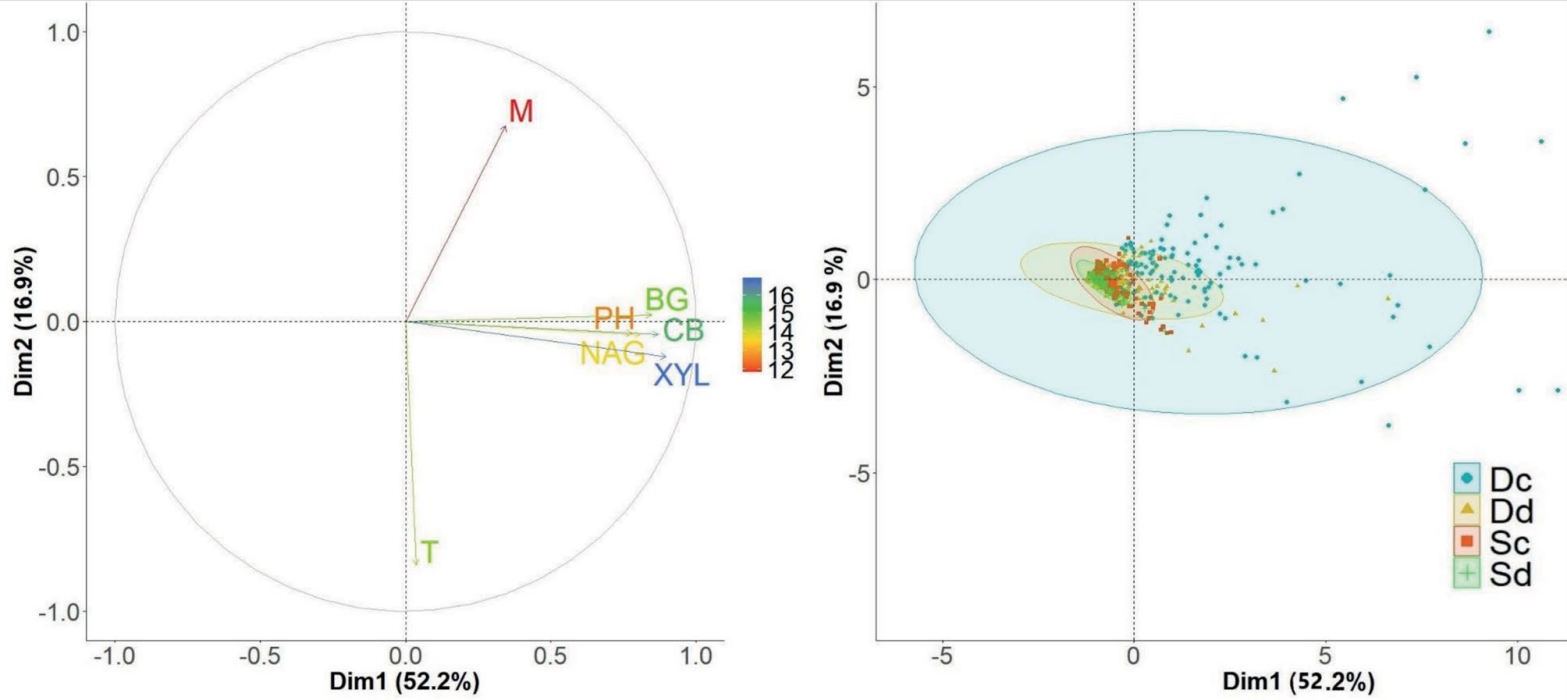
Principal Component Analysis (PCA) of enzyme activity and environmental variables in the deadwood–soil system. The left panel shows the projection of variables on the plane of the first two principal components, including β-D-cellobiosidase (CB), β-xylosidase (XYL), N-acetyl-β-D-glucosaminidase (NAG), β-glucosidase (BG), phosphatase (PH), moisture (M), and temperature (T). The right panel shows the distribution of samples according to sample type: Dc, deadwood under control; Dd, deadwood under drought; Sc, soil under control; and Sd, soil under drought.

The Spearman correlation analysis shows a statistically significant relationship between enzyme activity and moisture content (Fig. 8). The highest correlation coefficient was noted between PH activity and moisture content (0.27). The correlation coefficient between the remaining enzymes and moisture content was also statistically significant. No significant relationship was observed between the activity of the enzymes tested and temperature (Fig. 8). The conducted PCA analysis explains approximately 70% of the variability of the properties included in the analysis (Fig. 9). PCA analysis supported the association between enzyme activity and moisture. The PCA also indicated partial separation between deadwood and soil samples (Fig. 9). Factor 1 was primarily associated with enzyme activity, whereas factor 2 reflected variation related to moisture and temperature.

## Discussion

The conducted experiment showed that simulated drought significantly affected the activity of enzymes participating in the C, N and P cycle in decaying wood. In most cases (79–96% of observations), the activity of all measured enzymes decreased in wood subjected to simulated drought. The magnitude of these decreases varied among enzymes, usually intensifying with the duration of drought. Initially, the decrease in activity was smaller (in the case of various enzymes it was on average 10–40%) and then increased to 50–90%. In the scientific literature, the influence of drought on wood decay processes remains underexplored, yet Jia et al. (2024) demonstrated that reducing rainfall by 35% and 70% lowered carbon dioxide emissions from decaying wood by 28% and 48%, respectively, indicating diminished microbial activity during drought conditions. The wood dried out gradually, initially having a moisture content of 30–82% (depending on species); during the experiment, the moisture content of the logs decreased by 20–74%. Direct precipitation was effectively excluded by the experimental design, allowing only limited moisture input via lateral airflow and minor seepage from the soil surface. This approach enabled the isolation of moisture-driven effects on enzymatic activity while maintaining near-natural thermal conditions. When speaking about the decomposition of wood in contact with soil, it is important to account for the possibility of active water transport through the mycelium shreds that grow through the wood and are in contact with a more humid environment. Stienen et al.^33^ showed that the mycelium of selected basidiomycetes can develop in wood at very low humidity (2–17%), and that these fungi differ in their ability to transport water through their hyphae.

The observed decrease in extracellular enzyme activity under drought conditions indicates a functional suppression of microbial processes within deadwood. Fungal decomposers secrete a broad suite of enzymes involved in cellulose, hemicellulose and lignin degradation, including cellobiose dehydrogenase, β-glucosidase, endoglucanase, cellobiohydrolase, β-xylosidase, xylanase, xyloglucanase, acetyl esterases, and oxidative enzymes such as peroxidases and laccases^34^. In this study, we focused on three enzymes directly involved in wood polysaccharide decomposition (CB, BG, XYL) and two enzymes indirectly linked to nutrient cycling: NAG, reflecting chitin degradation and nitrogen mineralization^35^ and PH, responsible for organic phosphorus mineralization. Oxidative enzymes were not included in the present study because the experimental design focused on hydrolytic enzymes linked to C, N and P acquisition, which provide sensitive indicators of microbial nutrient demand and biochemical responses to moisture limitation. Therefore, the results should be interpreted as reflecting hydrolytic enzyme-mediated processes rather than the full enzymatic machinery of lignin degradation. These results suggest that drought may limit not only enzymes directly involved in wood polysaccharide decomposition, but also enzymatic processes linked to N and P acquisition in deadwood. These enzymatic patterns are consistent with our earlier findings from the same experimental site, where simulated drought significantly altered C/N/P stoichiometry and lignin dynamics in the deadwood–soil system^3^. In that study, drought widened C/N and C/P ratios and promoted the accumulation of lignin-derived carbon, suggesting constrained nutrient mobilization. Together, both studies indicate that drought-induced suppression of enzymatic activity represents a key mechanistic pathway linking moisture limitation to altered elemental stoichiometry and increased chemical recalcitrance of decomposing wood. Among the three enzymes, BG showed the strongest response to drought stress. Average differences in the wood of four species subjected to drought stress (except beech and spruce) exceeded 300 nmol MUB g^−1^ ·h^−1^. For CB and XYL, such differences exceeded 30 nmol MUB g⁻^1^·h⁻^1^ in half of the tested species. However, it must be considered that BG typically shows much higher activity in dead wood compared to CB and XYL^36^.

Drought-induced declines in enzyme activity varied markedly among wood species. All the enzymes involved in wood decomposition (CB, BG, XYL), as well as phosphatase (PH), showed a significantly smaller decrease in activity under drought in beech and spruce wood. It is difficult to clearly identify the reasons for such species-specific responses to drought stress. These species-specific responses may partly reflect differences in microbial functioning within decaying wood, as indicated indirectly by the observed patterns of extracellular enzyme activity. The presence of fungi better adapted to moisture deficits, capable of transporting water, or possessing higher enzymatic activity may increase the wood’s resistance to drought stress and thus limit the decline in enzymatic activity. In particular, filamentous fungi have the ability to actively transport water^37,38^.

Species-driven variability may also arise from initial wood properties. Although all logs were assigned to decay class IV^18^, wood of different species requires different decomposition durations to reach comparable structural and biochemical states. Differences in density, porosity and the proportion of incrusting compounds, particularly in heartwood, influence microbial colonization and enzymatic accessibility. As a result, wood with higher density or lower permeability may exhibit greater resistance to functional degradation under drought, even at similar decay stages. The role of wood physical and chemical traits in controlling decomposition dynamics has been widely documented^39–41^.

In addition to deadwood, we observed clear drought effects on enzymatic activity in soil directly underlying decaying logs. Although the study area was selected to minimize environmental heterogeneity, the observed responses have broader implications for understanding soil organic matter stabilization under moisture stress. Deadwood decomposition influences nutrient retention and microbial activity in adjacent soil, contributing to soil formation and ecosystem resilience^9,42^. The decrease in activity in the soil under deadwood occurred in 73–83% of observations and reached slightly lower values compared to the decrease observed in wood. For BG, these differences were in most cases >60 nmol MUB g^−1^ ·h^−1^, for PH >30 nmol MUB g^−1^ ·h^−1^, and for CB >4 nmol MUB. The decrease in the activity of the tested enzymes in the soil under wood should probably be associated with the limited transport of wood decomposition products to the soil with precipitation, which, by washing the logs, contributes to the leaching of a number of nutrients into the soil^42–45^.

The varying responses of wood enzyme activity may indicate the pace at which substances pass from deadwood into the soil. The observed changes in soil enzymatic activity may affect the microbial dynamics of the soil under deadwood. In particular, the processes of water transport by mycelium may be an adaptive mechanism that allows microorganisms to function under conditions of limited water availability^33^.

During the measurement cycle, we found fluctuations in the activity of the measured enzymes both in the case of wood subjected to drought stress and receiving precipitation. Enzyme activity generally increased during the first vegetation season and peaked during the summer months (June–September), with occasional high values also observed in early spring (March of the second year), followed by a decline toward winter. It should also be considered that part of the response observed during the first year may reflect microbial and biochemical adjustment to the presence of advanced-decay deadwood at the soil–wood interface, in addition to the precipitation exclusion treatment itself. The increase in the activity of the tested enzymes during the first season was probably associated with thermal conditions. The rapid increase in activity after the winter period may be associated with the release of substrates resulting from wood and soil thawing, as well as with increased lysis of dead microorganisms. In the second year (2024), the effects of drought on enzyme activity were generally more pronounced, particularly for β-D-cellobiosidase (CB) and β-glucosidase (BG), where larger contrasts between control and drought treatments were observed. This indicates a strengthening of moisture limitation effects over time, especially in deadwood, where activity reductions under drought reached their highest values. However, the response was not uniform across all enzymes, suggesting that drought effects were strongly modulated by species- and substrate-specific properties rather than a simple temporal trend. Under drought conditions, the activity of enzymes associated with organic carbon degradation (BG, CB) shows a marked decrease, indicating a slower rate of wood decomposition and a longer retention of carbon in the ecosystem. This may indicate that drought-related suppression of hydrolytic enzyme activity can temporarily slow decomposition processes in deadwood, although direct effects on carbon fluxes and long-term carbon storage were not measured in this study. Evidence for the existence of this type of phenomenon was confirmed in earlier studies^40,46^. Statistical analysis of the results of the activity of the tested enzymes and an attempt to correlate them with external factors such as temperature and humidity indicated a more significant effect of wood moisture on the activity level of the tested enzymes, especially PH and BG. The importance of soil moisture and habitat conditions for deadwood decomposition has been emphasized in previous studies^10,36,47^. Often, colder sites with stable or higher moisture create more favourable conditions for the development of microorganisms that decompose dead wood and the activity of their enzymes^10^. Our results indicate that enzymatic activity in soil under deadwood responds differently to drought compared to deadwood itself; however, these patterns should be interpreted with caution. Although smaller absolute changes were sometimes observed in soil, this may partly reflect its lower baseline activity rather than greater stability. Moreover, as our study did not include soil without deadwood as a control, we cannot directly assess the buffering effect of deadwood on soil microclimate or enzymatic processes under drought conditions. In our study, in the period when a rapid increase in enzyme activity was observed at the turn of the first winter and spring of 2024, a clear increase in the moisture content of wood subjected to drought stress was demonstrated. This was likely an important factor that, apart from the increase in easily decomposed organic compounds from dead microorganisms, influenced the rapid increase in the activity of the tested enzymes.

Several limitations should be considered when interpreting these results. First, the experiment did not include a soil-only control without deadwood, which limits direct inference about the isolated effect of deadwood on soil enzymatic activity under drought. Second, the study focused on hydrolytic enzymes related to C, N and P acquisition, while oxidative enzymes involved in lignin degradation were not measured. Third, the enzymatic assays were conducted under standardized laboratory conditions and should therefore be interpreted as comparative indicators of potential enzyme activity rather than direct measurements of in situ reaction rates. Finally, the statistical approach was designed to identify general patterns across treatments and sample types, rather than to fully model the hierarchical repeated-measures structure of the experiment.

## Conclusions

Under drought conditions, the activity of all enzymes tested in deadwood decreased significantly, often by more than 50% compared to control conditions, indicating strong moisture limitation of microbial functioning during advanced stages of wood decomposition. Enzymatic activity in soil beneath deadwood also declined under drought, although the observed changes were generally smaller in absolute terms than in deadwood. The magnitude of drought-related decreases was enzyme-specific and generally less pronounced in soil, particularly for β-glucosidase and β-xylosidase, where differences between control and drought treatments ranged from approximately 30–40%. In both substrates, enzymatic activity exhibited clear seasonal patterns, with highest values recorded in summer and lowest in winter. The stronger divergence between control and drought treatments observed in the second year of the experiment (2024) indicates cumulative effects of prolonged moisture deficit on enzymatic functioning in the wood–soil system. Our results demonstrate that drought-induced suppression of enzymes associated with organic carbon degradation (β-glucosidase and β-D-cellobiosidase) may slow down decomposition processes in deadwood, potentially extending carbon residence time at the local ecosystem scale. However, these effects should be interpreted as functional responses within the studied system rather than direct measures of carbon fluxes or long-term storage. Overall, this study highlights the sensitivity of enzymatic processes in decomposing deadwood to sustained moisture limitation and emphasizes the contrasting responses of wood and adjacent soil. By capturing drought-driven changes in key extracellular enzymes, the results provide process-level insight into how biochemical functioning at the deadwood–soil interface responds to increasing drought frequency, informing assessments of future decomposition dynamics in temperate forest ecosystems.

## Supplementary Information

Supplementary Information.

## Data Availability

The datasets generated and analysed during the current study are available from the corresponding author, Adam Górski (adam.gorski@student.urk.edu.pl), upon reasonable request.
