# Supplementary figures and images for: Enzymatic indicators reveal drought sensitivity of the deadwood–soil system in temperate forests
Source: Sci Rep. 2026 May 23;16:23685. doi: 10.1038/s41598-026-54208-6 (PMC13424347; doi:10.1038/s41598-026-54208-6)

■ DC ■ DD

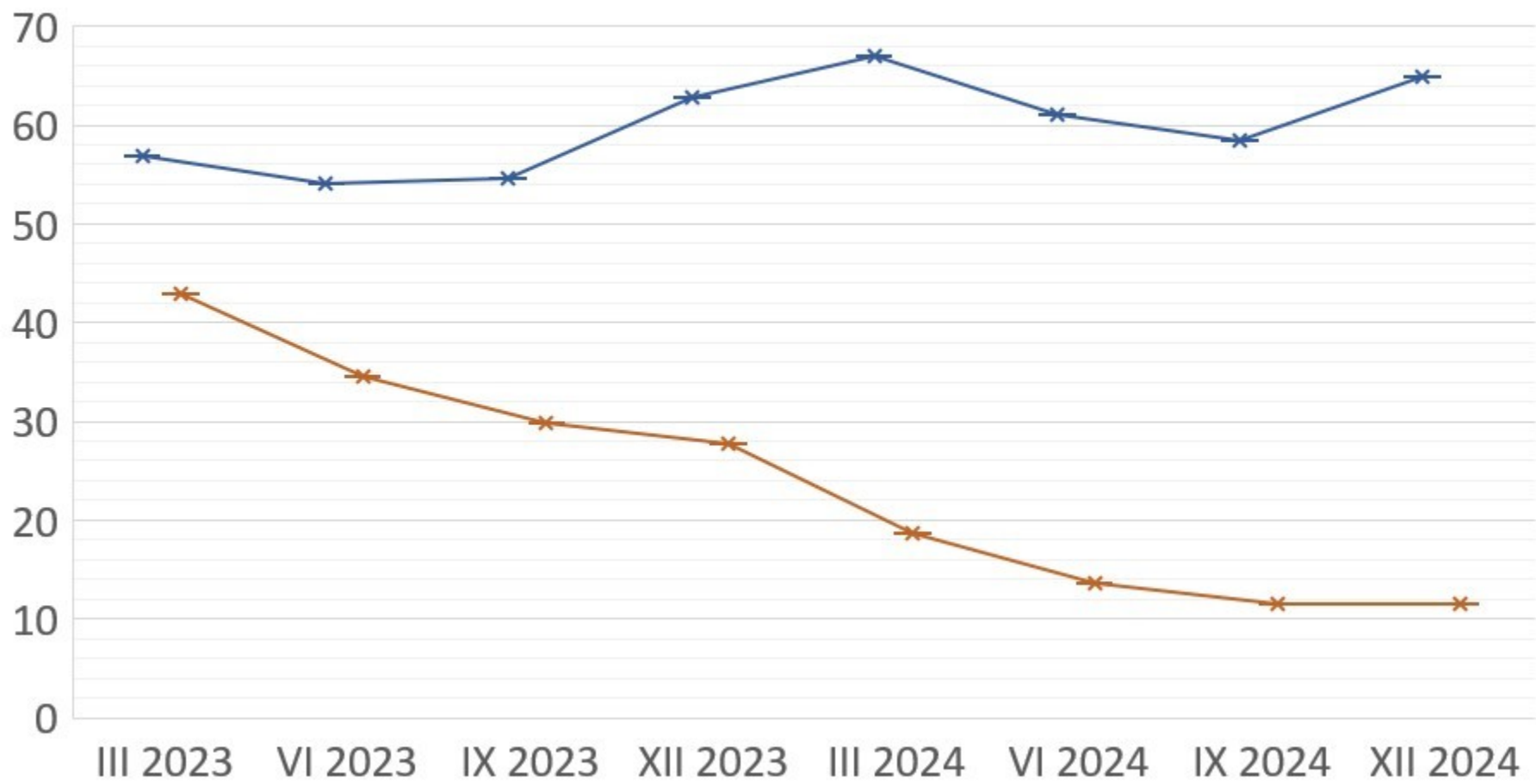

Supplement: Supplementary file 1 — Supplementary Information. [file 41598_2026_54208_MOESM1_ESM.pdf]
